# A Singular Case

**Published:** 1881-08

**Authors:** 


### EDITORIAL.

### A SINGULAR CASE.

Dr. A. E. Emminger, of Brazil, Indiana, in a private letter, gives the following uncommon and interesting case. He says: "The patient, a lady, presented herself for an examination and treatment of the mouth and teeth. I found the following conditions, viz.:

"1st. Salivary calculus in large quantities in some parts of the mouth, from which offensive odor proceeded.

"2d. She had several badly decayed teeth, two of which were pulpless.

"3d. She stated that about three years ago her mouth became inflamed, swollen and abscesses were formed at several different places in the mouth, notwithstanding there were only two teeth in the mouth with dead pulps.

"How could these abscesses come without more of the teeth being devitalized?

"My treatment consisted in the removal of all deposit of foreign material of any kind, and treatment of the gums to reduce inflammation and to give tone to the tissue.

"The patient has just made her appearance after an absence of three or four weeks, with the mouth very greatly improved. How did the abscesses originate?"

The abscesses referred to here were probably not from the sockets of the teeth, but arose in the soft tissue, and resulted from irritation by salivary calculus, of a structure particularly predisposed to inflammation; then, of course, the removal of the calculus with some mild appropriate treatment would be sufficient for restoration, or just the result that did take place in this instance.

